# Alterations in the Plasma Levels of Specific Choline Phospholipids in Alzheimer’s Disease Mimic Accelerated Aging

**DOI:** 10.3233/JAD-171036

**Published:** 2018-02-20

**Authors:** Fabian Dorninger, Ann B. Moser, Jianqiu Kou, Christoph Wiesinger, Sonja Forss-Petter, Andreas Gleiss, Margareta Hinterberger, Susanne Jungwirth, Peter Fischer, Johannes Berger

**Affiliations:** a Department of Pathobiology of the Nervous System, Center for Brain Research, Medical University of Vienna, Vienna, Austria; b Peroxisomal Diseases Laboratory, The Hugo W Moser Research Institute, The Kennedy Krieger Institute, Baltimore, MD, USA; c Center for Medical Statistics, Informatics, and Intelligent Systems, Medical University of Vienna, Vienna, Austria; d Ludwig Boltzmann Institute of Aging Research, Danube Hospital, Vienna, Austria; e Department of Psychiatry, Medical Research Society Vienna D.C., Danube Hospital, Vienna, Austria

**Keywords:** Biomarkers, blood, lyso-PAF, lysophosphatidylcholines, lysophospholipids, mass spectrometry, plasmalogens

## Abstract

Alzheimer’s disease (AD) is the most common neurodegenerative disease and of continuously rising prevalence. The identification of easy-to-measure biomarkers capable to assist in the prediction and early diagnosis of AD is currently a main research goal. Lipid metabolites in peripheral blood of human patients have recently gained major attention in this respect. Here, we analyzed plasma of 174 participants (not demented at baseline; mean age: 75.70±0.44 years) of the Vienna Transdanube Aging (VITA) study, a longitudinal, population-based birth cohort study, at baseline and after 90 months or at diagnosis of probable AD. We determined the levels of specific choline phospholipids, some of which have been suggested as potential biomarkers for the prediction of AD. Our results show that during normal aging the levels of lysophosphatidylcholine, choline plasmalogen, and lyso-platelet activating factor increase significantly. Notably, we observed similar but more pronounced changes in the group that developed probable AD. Thus, our results imply that, in terms of choline-containing plasma phospholipids, the conversion to AD mimics an accelerated aging process. We conclude that age, even in the comparatively short time frame between 75 and 82.5 years, is a crucial factor in the quest for plasma lipid biomarkers for AD that must be carefully considered in future studies and trials.

## INTRODUCTION

Alzheimer’s disease (AD) is the most prevalent neurodegenerative disorder and the most common form of dementia. Affected individuals suffer from pronounced cognitive decline including memory loss, confusion, mood and personality changes, and, eventually, inability to manage everyday life. The detailed molecular mechanism underlying the disease is still unknown and numerous factors have been suggested to contribute to pathology [1]. Advanced age represents the greatest risk factor for AD. In addition to the three genes (*APP*, *PSEN1,* and *PSEN2*) associated with the rare familial (early onset) variants, genetic factors also modulate the risk of developing the common sporadic forms of AD, with the *ɛ*4 allele of the *apolipoprotein E* (*APOE*) gene being the most important so far [2].

The high prevalence of AD poses a major health care problem on society. Currently, more than 35 million people worldwide are affected by AD or other types of dementia [3]. As longevity increases, this number is predicted to increase dramatically in the next decades; for example, prognoses for the United States assume nearly a tripling of AD cases by 2050 [4]. In order to deal with these imminent challenges, it is imperative to clarify the etiology of AD and to identify biomarkers that allow easy diagnosis of the disease at preclinical stages. Currently, most biomarkers in use are related to the classical neuropathological hallmarks of AD, including determination of the levels of tau and amyloid-*β* (A*β*) in cerebrospinal fluid, positron emission tomography (PET) imaging of amyloid deposits, and magnetic resonance imaging (MRI) for evaluation of brain atrophy [5]. However, these analyses are costly and involve elaborate, partly invasive, medical procedures. Due to the simplicity and low burden for patients, reliable blood-based biomarkers would offer a highly attractive alternative. Recently, several studies have reported progress in the search for plasma or serum markers that could be of value in the diagnosis or even prediction of AD [6–10]. Among others, altered levels of circulating phospholipids have been associated with the development of AD [7, 11, 12]. Certain choline phospholipid species have been proposed as biomarkers for the prognosis [7] and diagnosis [13] of AD, but reproducibility of biomarker studies involving these phospholipids appears to be a major issue [14] and only limited information is available on the development of their plasma levels derived from longitudinal studies. In addition, our previous findings indicated an impairment of peroxisomes—small, metabolically highly versatile organelles [15]—in the brain of AD patients [16].

Here, we aimed to combine and extend these observations to the systemic level by examining changes in the plasma levels of selected choline-containing phospholipids, whose metabolism involves peroxisomes, in the course of conversion to probable AD and comparing these with the alterations during normal aging. The Vienna Transdanube Aging (VITA) study, a prospective population-based birth cohort study with highly standardized conditions of participation [17], sets an optimal stage for addressing fluctuations in plasma choline phospholipids over time. All individuals entering the VITA study were enrolled from two districts of the city of Vienna, Austria, at the age of about 76 years and were monitored over the following 90 months (7.5 years), an age range when the incidence of AD increases considerably [2]. All the included participants had been cognitively tested and found not demented at study entrance (baseline). During the following 90 months, as expected, a part of the cohort converted to probable AD, whereas the majority remained cognitively intact without any signs of dementia. This setting, thus, allowed us to discriminate between phospholipid alterations occurring during normal aging and those related to the development of AD.

## MATERIALS AND METHODS

### Study design and sample cohorts

A detailed description of patient recruitment, inclusion criteria and participation in the VITA study has been published previously [17]. Briefly, all 606 participants lived in the 21st and 22nd district of Vienna, Austria, and were enrolled around their 76th birthday (mean age whole group: 75.77±0.45 years). Extensive testing at baseline and after 30 and/or 60 and/or 90 months included fasting blood collection, psychiatric and neurological examination, depression analysis, complete demographic, psychosocial, and medical history including current and former drugs and diseases using a structured interview. Cranial MRI was performed using a 1.0 Tesla unit (Siemens Impact Expert). The following sequences were obtained: transverse proton density and T2-weighted turbo spin echo, coronary T1-weighted gradient echo sequence (MPRAGE) and a thin-section inversion recovery sequence in the olfactory region. For the rating of medial temporal lobe atrophy, the hippocampal area along the longitudinal axis of the hippocampus was reconstructed. Atrophy was evaluated using a scoring scheme ranging from 0 (normal/no atrophy) to 4 (most severe atrophy). Each person underwent neuropsychological testing according to the CERAD (Consortium to Establish a Registry for Alzheimer’s Disease) protocol [18]. In addition, several tests for memory (e.g., Fuld Object Memory Evaluation, FOME) and for cognitive processing speed and executive function (Trail Making Tests (TMT) A & B) were done. Whenever possible, a close relative was interviewed in addition. At all investigations, dementia and depression were diagnosed by a psychiatrist according to the common criteria of the Diagnostic and Statistical Manual of Mental Disorders (DSM-IV). Clinical and laboratory information on the study population are summarized in Table 1 and Supplementary Table 1. The NINCDS-ADRDA [19] diagnoses of possible and probable AD were made in consensus conferences using all available information about psychometric results, clinical investigation, and MRI data. Subjects with mild cognitive impairment were always classified with the non-demented. Twenty persons were already demented, 586 were not demented at baseline. In the present investigation, we included only those participants who were not demented at baseline, had plasma samples frozen at baseline and at the 90-month follow-up visit and either remained non-demented (control group; *n* = 152; 58 males, 94 females; mean age at baseline: 75.68±0.43 years and at 90 months: 83.28±0.44 years) or who were diagnosed with probable AD during the 90-month follow-up period (*n* = 14 at 30 months, *n* = 4 at 60 months, *n* = 4 at 90 months) and had plasma samples frozen at baseline and at the time of diagnosis (probable AD group; *n* = 22; 7 males, 15 females; mean age at baseline: 75.80±0.49 years and at diagnosis: 79.71±1.99 years). Persons with a diagnosis of possible AD were not included in this study because of the observation of reversibility and, therefore, uncertainty of this diagnosis [20]. Samples of the control group and the AD group taken at baseline were compared with those at 90 months and at diagnosis of probable AD, respectively. Four baseline samples of the control group had to be excluded from the data set of choline plasmalogen (PlsCho) and lyso-platelet activating factor (lyso-PAF) but not lysophosphatidylcholine (lysoPC) levels due to technical issues. All participants signed informed consent, as approved by the Ethics Committee of the City of Vienna, and all procedures were performed in accordance with the guidelines stipulated in the Declaration of Helsinki (1975). Determination of phospholipids in the plasma samples was done in a blinded fashion.

**Table 1:** Description and major diagnostic parameters of the study population

	Baseline		Follow-up		Statistical comparisons (*p* values)			
	Aging (*n* = 152; 94 F, 58 M)	AD (*n* = 22; 15 F, 7 M)	Aging (*n* = 152; 94 F, 58 M)	AD (*n* = 22; 15 F, 7 M)	Aging BL versus AD BL^*^	Aging FU versus Aging BL^†^	AD FU verrsus AD BL^†^	Aging FU versus AD FU^‡^
Age [y]	75.64	75.79	83.22	78.85	n.d.	n.d.	n.d.	n.d.
	(75.32–75.98)	(75.44–76.22)	(82.95–83.52)	(78.08–80.92)				
MMSE	28.0	26.5	28.0	21.5	0.001	0.710	<0.001	<0.001
[0–30 points]	(27.0–29.0)	(24.0–29.0)	(27.0–29.0)	(19.75–24.0)				
FOME	45.0	38.5	44.0	23.0	0.001	0.163	<0.001	<0.001
[0–50 points]	(43.0–47.0)	(35.0–46.0)	(41.0–47.0)	(13.75–28.0)				
TMT-A^§^	43.5	58.0	53.0	108.0	<0.001	0.006	<0.001	<0.001
[s]	(36.0–54.3)	(48.0–78.5)	(42.0–71.0)	(71.8–208.3)				
TMT-B^§^	117.0	172.0	170.0	600.0	<0.001	<0.001	<0.001	0.001
[0–600 s]	(89.0–151.0)	(145.0–235.0)	(110.5–310.0)	(487.5–600.0)				
Brain MRI^§^	0:93.0%	0:47.1%	0:31.8%	0:0%				
Scores^¶^	1:6.3%	1:35.3%	1:42.4%	1:23.5%				
[% per group]	2:0.7%	2:17.6%	2:18.2%	2:52.9%	0.004	<0.001	<0.001	0.007
	3:0%	3:0%	3:7.6%	3:11.8%				
	4:0%	4:0%	4:0%	4:11.8%				

BL, baseline; FU, follow-up; F, female; M, male; MMSE, Mini-Mental State Examination; FOME, Fuld Object Memory Evaluation; TMT, Trail Making Test. All values (except for brain MRI scores) are presented as medians with quartiles in parentheses. Follow-up refers to 90 months in the aging group and the time point of diagnosis in the AD group. ^*^Baseline comparisons were done using Wilcoxon’s rank sum test (brain MRI: Fisher’s exact test). ^†^Longitudinal changes in the normal aging and the AD group were evaluated using ANCOVA models (brain MRI: McNemar’s test). ^‡^Comparison of the longitudinal changes in the aging versus AD group was carried out using ANCOVA models (brain MRI: logistic regression). ^§^Number of missing values: TMT-A: aging group: 2 at BL, 2 at FU; AD group: 1 at BL; TMT-B: aging group: 5 at BL, 7 at FU; AD group: 7 at BL, 1 at FU; Brain MRI: aging group: 10 at BL, 20 at FU; AD group: 5 at BL, 5 at FU. ^¶^Brain atrophy in MRI was scored from 0 (no atrophy) to 4 (most severe atrophy). Data are shown as relative frequencies in each group.

### Chemicals and reagents

Methanol (Optima^TM^ LC/MS) was obtained from Thermo Fisher and GC/MS grade water and chloroform were obtained from Burdick & Jackson; ammonium formate and formic acid were from Sigma-Aldrich. All mobile phase mixtures were filtered through a 0.2*μ*m Nylon 66 membrane (Supelco) before use. The internal standard ^2^H_4_-lysoPC C26:0 and the reference standards lysoPC C16:0, lyso-PAF C16:0, lysoPC C18:0, lyso-PAF C18:0, lysoPC C18:1, lysoPC C20:0, lysoPC C22:0, lysoPC C24:0, lysoPC C26:0, and PlsCho 18:0p/18:1, PlsCho 18:0p/20:4 and PlsCho 18:0p/22:6 were purchased from Avanti Polar Lipids. The internal standard ^2^H_4_-lyso-PAF C16:0 was purchased from Cayman Chemical. Screw-capped glass culture tubes with Teflon lined screw caps (13×100 mm) were obtained from Thermo Fisher and Verex 9 mm, polypropylene, 300*μ*l injection vials with PTFE/Silicone, pre-slit caps, were obtained from Phenomenex (part #ARO-9992-13). Nylon centrifuge tube filters (0.22*μ*m) with polypropylene tube Costar #8169 from Corning were purchased from Thermo Fisher and the HPLC column, a Zorbax Eclipse XDB-C8 column (4.5×50 mm, particle size: 3.5*μ*m), was obtained from Agilent Technologies.

### Plasma collection

All plasma samples were collected at 8 a.m. after overnight fasting and without prior intake of the morning doses of current medications. Blood was collected into polypropylene tubes containing K^+^-EDTA, kept on ice, centrifuged within 60 min, and stored at -80°C. The frozen samples were thawed once for preparation of aliquots and subsequently stored at –80°C until analysis. All transport processes were carried out on dry ice.

### Lipid extraction

Duplicate 10*μ*l aliquots of plasma samples were measured by pipetting into 13×100 mm screw-capped test tubes. To each, 150*μ*l of a methanol solution containing 15.6 pmol ^2^H_4_-lysoPC C26:0 and 20.6 pmol ^2^H_4_-lyso-PAF C16:0 were added. The tubes were closed with Teflon-lined screw caps and placed in a 37°C shaker bath for 1 h. The solution in each tube was transferred by pipet to 0.22*μ*m Nylon centrifuge tube filters and spun in an IEC Micromax RF centrifuge (International Equipment Company) at 14,000 rpm for 3 min at room temperature. The filtrate was collected by pipet and transferred to Verex 9 mm, polypropylene, 300*μ*l injection vials with PTFE/Silicone, pre-slit caps. The vials were stored at –20°C until analysis.

### Determination of phospholipids

The ultra-fast liquid chromatography tandem mass spectrometry (UFLC-MS/MS) measurements were carried out using a Shimadzu SIL-20AC UFLC system and an Applied Biosystems 3200 MS/MS mass spectrometer. The analyses of lysoPC are a modification of the method of Hubbard and colleagues [21]. The analyses of plasmalogens are modifications of the methods of Norris and colleagues and Hui and colleagues [22, 23]. Phospholipids were separated on a Zorbax Eclipse XDB-C8 column using a 6-minute gradient of 25% mobile phase (MP) B at 1 min to 100% MPB at 3.5 min, 100% MPB at 5.0 min to 25% MPB at 5.5 min (MPA = methanol:water:formic acid, 54.5:45:0.5 containing 2 mM HCOONH_4_; MPB = formic acid:methanol:chloroform, 0.5:89.5:10 containing 2 mM HCOONH_4_). Flow rate was 0.5 ml/min. Positive ion electrospray mass spectrometry with multiple reaction monitoring (MRM) and a dwell time of 100 ms per transition was used for each analyte. The following MRM transitions were monitored for the lysoPC species: C16:0, *m/z* 496>104; C18:2, *m/z* 520>104; C18:1, *m/z* 522>104; C18:0, *m/z* 524>104; C20:0, *m/z* 552>104; C22:0, *m/z* 580>104; C24:0, *m/z* 608>104; C26:0, *m/z* 636>104; ^2^H_4 - _C26:0, *m/z* 640>104; and for the lyso-PAF species: ^2^H_4_-C16:0, *m/z* 486>104; C16:0, *m/z* 482/104; C18:1, *m/z* 508>104; and C18:0, *m/z* 510>104. Due to the large number of analytes, a second injection was made for the plasmalogen analyses using the same column, mobile phases and gradient. The following MRM transitions were monitored for PlsCho species: 18:0p/18:1, *m/z* 772.7>184; 18:0p/22:6, *m/z* 818.64>183.9; 18:0p/20:4, *m/z* 794.66>184. The internal standard used for PlsCho was ^2^H_4_-lysoPC C26:0, *m/z* 640>184.

### Calculations and method validation

Standard curves were prepared by serial dilution from stock solutions for each lysoPC species and for each plasmalogen species, for which standards were available. If no standard was available, one point calibration was used. The lower and upper limits of quantitation were determined for each analyte. Two separate standard curves, the first containing three different concentrations of lysoPC mixtures and the second containing three different concentrations of plasmalogens were assayed three times on separate days for each new batch of methanolic extraction solutions. Accuracy and precision were determined and the coefficient of variation was calculated.

The retention time of each known standard peak was recorded. Peaks from standards, known and unknown samples were reviewed using the Analyst peak review quantitation program (AB Sciex). Peaks were identified by MRMs and retention times of standards. Data for each standard, known and unknown sample were processed and transferred to Excel using the programs in Analyst.

For quality control, plasma pools from normal (from the Red Cross), abnormal 1 (from leftover samples from individuals with X-linked adrenoleukodystrophy) and abnormal 2 (from leftover samples from plasmalogen-deficient patients) individuals were analyzed with each set of unknown samples. The coefficient of variation was calculated for each plasma pool. The plasma pools and study samples were assayed in duplicate and results were averaged.

Additional experiments after the original analyses revealed the presence of isobaric species in the peaks representing PlsCho species. We then performed control measurements involving plasma samples from plasmalogen-deficient patients and determined the acid lability of the lipid species represented by the respective peaks. Based on these data, we calculated the following maximal fractions accounted for by the isobaric species: *m/z* 772.7:<10%; *m/z* 818.64:<4%; and *m/z* 794.66:<6%. Other lipid species were not affected.

Total levels of lysoPC, PlsCho, and lyso-PAF were calculated by adding the values obtained for the levels of the individual subspecies analyzed.

### Determination of standard laboratory parameters and lipid-lowering medication

Total cholesterol, high density lipoprotein cholesterol (HDL), and triglycerides were analyzed with a Hitachi 917 analyzer (Roche) using reagents from the same manufacturer and low density lipoprotein cholesterol (LDL) was calculated.

Information on drug exposure was obtained from study participants: 97/152 control subjects and 16/22 subjects later diagnosed with probable AD did not use any lipid- or cholesterol-lowering drugs (including statins and fibrates) at any of the time points investigated. All others took lipid-lowering medication at least at one time point and were, consequently, excluded from the analysis of HDL, LDL, and triglyceride levels.

### Determination of APOE genotype

The presence of particular *APOE* alleles was determined for all participants by PCR using the LightCycler APOE assay kit (Roche). Individuals were either assigned to the group of subjects with at least one *APOE4* allele (controls: 31/152, AD: 11/22) or to that of subjects without an *APOE4* allele (controls: 121/152, AD: 11/22).

### Statistical analysis

**Fig. 1:**
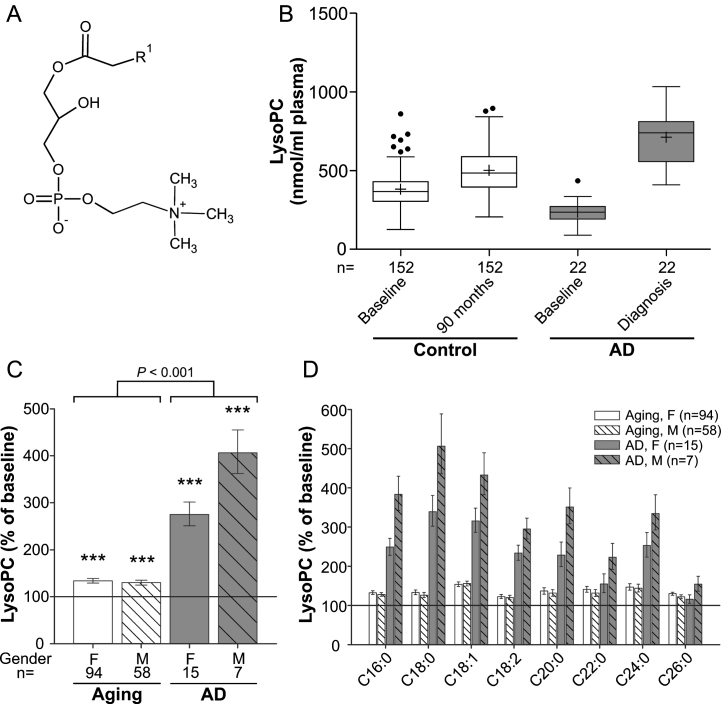
LysoPC levels in the plasma upon aging and conversion to AD. A) General structure of lysoPC (all lipid structures are drawn irrespective of stereochemistry). R^1^ is usually a saturated, mono- or dienoic fatty acyl residue. B) The distributions of total lysoPC values in plasma at baseline (76 years) and after 90 months (aged controls) or at diagnosis of probable AD after 30, 60, or 90 months (AD patients) are depicted as box-and-whisker plots according to Tukey’s method. The horizontal line inside the box indicates the median value, while + indicates the mean value. C) The intra-individual changes in total lysoPC levels during 90 months (aged controls) or up to probable AD diagnosis (AD patients) were calculated; the results are shown as percentage (geometric mean and standard error) of the baseline values (^***^p≤0.001 compared with baseline). The *p* value indicated above the brackets represents the comparison between aged control and AD patient groups according to ANCOVA models. D) Changes in individual lysoPC species grouped according to their *sn-*1 chain are depicted as in (C). F, female; M, male.

**Fig. 2:**
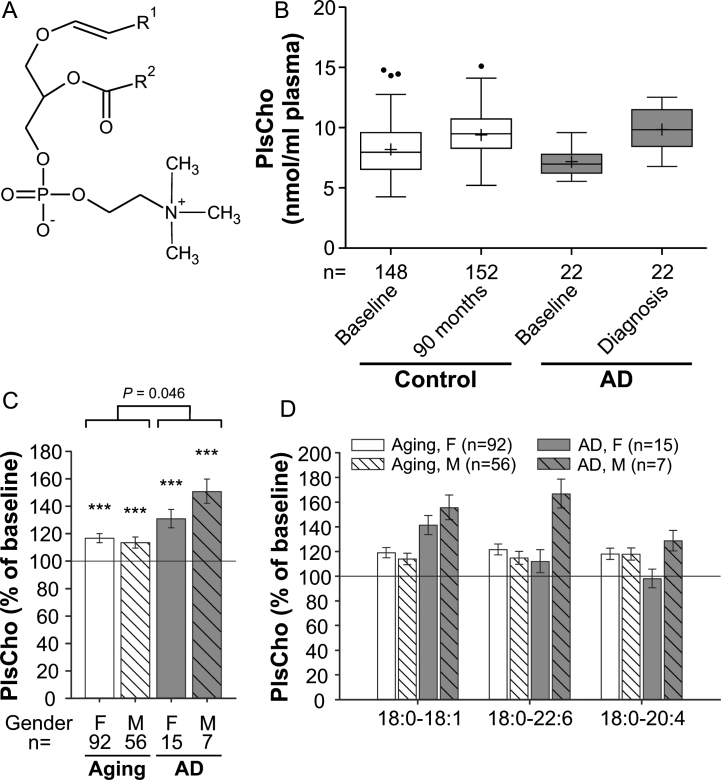
PlsCho levels in the plasma upon aging and conversion to AD. A) General structure of PlsCho. R^1^ is usually derived from C16:0, C18:0 or C18:1 fatty alcohols. R^2^ can be various fatty acyl residues. B) The distributions of total PlsCho values in plasma at baseline (76 years) and after 90 months (aged controls) or at diagnosis of probable AD after 30, 60, or 90 months (AD patients) are depicted as box-and-whisker plots according to Tukey’s method. The horizontal line inside the box indicates the median value, while + indicates the mean value. C) The intra-individual changes in total PlsCho levels during 90 months (aged controls) or up to probable AD diagnosis (AD patients) were calculated; the results are shown as percentage (geometric mean and standard error) of the baseline values (^***^p≤0.001 compared with baseline). The *p* value indicated above the brackets represents the comparison between aged control and AD patient groups according to ANCOVA models. D) Changes in individual PlsCho species grouped according to their *sn-*1 and *sn*-2 chains are depicted as in (C). F, female; M, male.

**Fig. 3:**
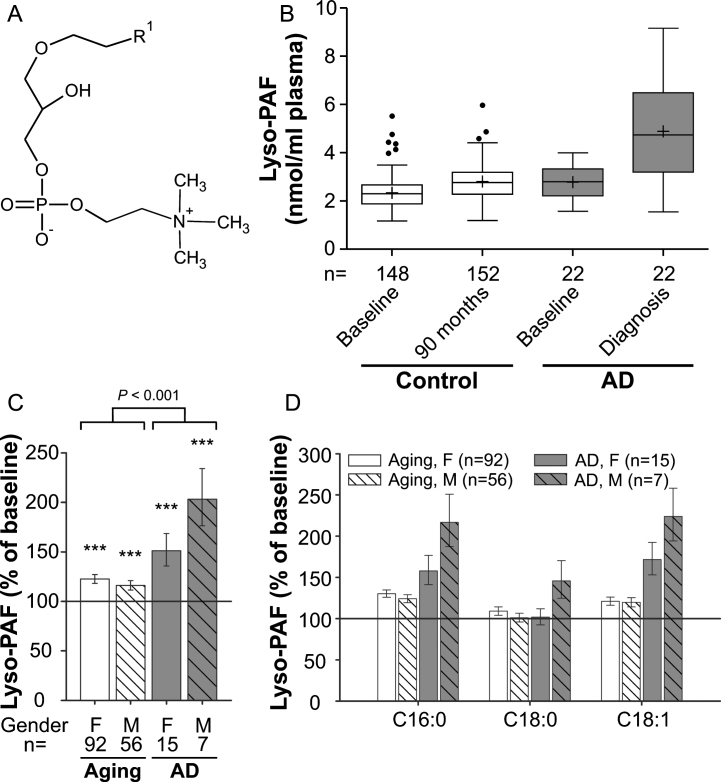
*Lyso-PAF levels in the plasma upon aging and conversion to AD. *A) General structure of lyso-PAF. R^1^ is usually derived from C16:0, C18:0 or C18:1 fatty alcohols. B) The distributions of total lyso-PAF values in plasma at baseline (76 years) and after 90 months (aged controls) or at diagnosis of probable AD after 30, 60, or 90 months (AD patients) are depicted as box-and-whisker plots according to Tukey’s method. The horizontal line inside the box indicates the median value, while + indicates the mean value. C) The intra-individual changes in total lyso-PAF levels during 90 months (aged controls) or up to probable AD diagnosis (AD patients) were calculated; the results are shown as percentage (geometric mean and standard error) of the baseline values (^***^p≤0.001 compared with baseline). The *p* value indicated above the brackets represents the comparison between aged control and AD patient groups according to ANCOVA models. D) Changes in individual lyso-PAF species grouped according to their *sn*-1 chain are depicted as in (C). F, female; M, male

Due to the right-skewed distributions of phospholipid levels, data were log-transformed to achieve approximately normally distributed residuals in the models described below. Levels of phospholipids are presented as box-and-whisker plots according to Tukey’s method or as bar charts with bars representing geometric means and error bars representing standard errors (calculated on the log scale and back-transformed asymmetrically to the original scale). Potential group differences with respect to log-transformed phospholipid ratios relative to baseline were investigated in analysis of covariance (ANCOVA) models with the group indicator as factor and the log-transformed baseline values as co-variable. The potential modulation of the group effect by gender (or another variable) was investigated by including gender (or the respective other variable) as well as its interaction with the group indicator into the model. Tests of phospholipid ratios against 100% were performed within the models described above adjusted within species (male/female, aging/AD group) using the method of Bonferroni-Holm. No further correction for testing multiple outcomes was done due to the exploratory character of the study. One ANCOVA model was estimated for each Figs. 1–3C, i.e., for each class of phospholipid. Baseline values for phospholipids were compared between groups using *t*-test applied on the log-scale. Associations between different types of phospholipid ratios were quantified using Spearman’s correlation coefficients.

To compare changes of psychometric test results (Mini-Mental State Examination (MMSE), FOME, TMT-A, TMT-B) from baseline to 90 months/diagnosis between groups, ANCOVA models analogous to those used for phospholipids were used. In these models, log-transforms of TMT-A and TMT-B values were used to obtain approximately normally distributed residuals, whereas MMSE scores were used as log(30-MMSE+1) due to its left-skewness. Group-specific changes were tested within these models. Comparisons of baseline values were done using Wilcoxon’s rank sum test (Fisher’s exact test for brain MRI scores). Change of brain MRI scores was investigated by a logistic regression model for categories 0+1 versus 2+3+4 including baseline values as model term in addition to gender. Firth’s method [24] was used due to separation problems. McNemar’s test was used to test changes within each group. Two-sided *p* values ≤0.05 were considered to indicate statistical significance. Upon presentation of clinical and laboratory parameters (cognitive measures and blood parameters), median values and quartiles are given due to the right-skewness of the data. Quartiles were calculated using the weighted means method. All calculations were done using SAS 9.4 (SAS Institute Inc.) or SPSS Statistics 21 (IBM).

## RESULTS

### Role of modulating factors

In the course of the VITA study, we analyzed a variety of choline-containing phospholipid species in the plasma of elderly human controls and originally non-demented individuals who later converted to AD. Peripheral blood samples were collected at baseline (mean age±SD: controls: 75.68±0.43 years; probable AD group: 75.80±0.49) and again 90 months later (mean age: 83.28±0.44 years) or, if earlier, at diagnosis of probable AD (according to NINCDS-ADRDA criteria; mean age: 79.71±1.99 years). Cognitive measures and brain MRI differed slightly, but statistically significantly, between the groups already at baseline (Table 1) and worsened markedly until follow-up in the AD group, whereas in the normal aging group much smaller changes were observed. Biochemical blood parameters obtained at baseline and follow-up visits (Supplementary Table 1) revealed no alternative physiological cause for cognitive deficits. First, the influence of the following, potentially modulating factors on the collected lipid data was examined: gender, *APOE* genotype, and intake of lipid-lowering drugs. Statistical evaluation using ANCOVA revealed neither a major impact of the latter two factors on the levels of analyzed phospholipids nor any modulation of the group effect. However, we found pronounced differences in various parameters between the sexes. Accordingly, most of the lipid analyses are depicted separately for males and females. For the analyses of HDL, LDL, and triglycerides, the data from individuals taking lipid-lowering drugs were excluded.

### LysoPC levels increase moderately during aging and are strikingly elevated in AD patients

LysoPC (1-acyl-*sn*-glycero-3-phosphocholines; Fig. 1A) are proinflammatory lipid mediators produced by hydrolysis of phosphatidylcholine (PC) and can constitute up to 20% of total plasma phospholipids in mammals [25]. Upon analysis of the major lysoPC species (Supplementary Figure 1A), as demonstrated by the distribution of the values at baseline and after 90 months (Fig. 1B), we observed a small (∼30%) but statistically highly significant increase in total lysoPC levels during normal aging (ANCOVA: F = 91.12, *p* < 0.001; Fig. 1C) and both sexes were similarly affected. In the group that developed probable AD, total lysoPC levels were lower than in controls at baseline (*p* < 0.001) but later, after onset of clinical AD, had increased to substantially higher levels than in the non-demented aging controls (Fig. 1B). The average intra-individual increases amounted to 175% in females and 306% in males of the probable AD group (mean increase across genders: aging group: 32%, AD group: 211%; group comparison using ANCOVA: *p* < 0.001; Fig. 1C). The pattern of elevations in plasma lysoPC of the control as well as the probable AD group was similar for all abundant lysoPC species measured (Fig. 1D). Noteworthy, this applied also to the lysoPC species 18:2 (Supplementary Figure 1B), which was recently included in a panel of lipid biomarkers claimed to predict conversion to AD [7]. We observed lower levels of lysoPC 18:2 at baseline in the future AD converters compared with controls. However, as for total lysoPCs, we detected a remarkable increase after diagnosis of the disease.

Moreover, we were particularly interested in the development of the levels of lysoPC 24:0 and lysoPC 26:0, as these are indicators of the degradation of very long-chain fatty acids, which happens in peroxisomes. Similarly to the other determined lysoPC species, the amounts of lysoPC 24:0 and 26:0 in the plasma were elevated upon aging and, even more, upon development of AD, but the increases were not more pronounced than for the other lysoPC species (Fig. 1D).

Our study likely even underestimates the difference in the rate of increase between normal aging and AD; because for the majority of AD patients, diagnosis of the disease (and blood sampling for this study) occurred prior to the 90-month follow-up visit of the non-demented controls (Table 1).

### Increases in PlsCho levels found in normal aging are more pronounced in AD patients

Several studies have previously identified plasmalogens as potential biomarkers in AD [11, 26]. Plasmalogens (1-(1Z-alkenyl), 2-acylglycerophospholipids) belong to the group of ether-linked phospholipids, which require peroxisomes for their biosynthesis. They are characterized by a vinyl ether bond at their *sn-1* position and have been implicated in a variety of cellular functions [27, 28]. Most plasmalogens harbor either choline or ethanolamine as a head group. Previous AD research has mainly focused on ethanolamine plasmalogens (1-(1Z-alkenyl),2-acylglycerophosphoethanolamines; PlsEtn), which were shown to be reduced in various brain regions and peripheral blood of patients [16, 29, 30]. However, as PlsEtn and PlsCho (1-(1Z-alkenyl), 2-acylglycerophosphocholines; Fig. 2A) differ in their chemical and biophysical attributes [31], we analyzed PlsCho levels in plasma at baseline (Supplementary Figure 2), and upon aging and conversion to AD.

Interestingly, the PlsCho levels obtained in our study showed the opposite pattern of that reported for PlsEtn. We noticed a highly statistically significant increase of 15% in total PlsCho levels of the normally aged cohort at the 90-month follow-up visit compared with the baseline values (ANCOVA: F = 147.49, *p* < 0.001; Fig. 2B,C). The PlsCho levels differed only marginally between the control and probable AD groups at baseline (*p* = 0.049), but the intra-individual increase over time was stronger in individuals developing probable AD (mean increase across genders: aging group: 15%, AD group: 37%; group comparison using ANCOVA: *p* = 0.046). By analogy with lysoPC, total PlsCho levels increased more strongly in male than in female AD patients reaching 151% and 131% of the baseline values, respectively (Fig. 2C). Particularly in males, the elevation in PlsCho was reflected by all three examined species in a similar pattern (Fig. 2D), again emphasizing that the changes in AD mimic those occurring during normal aging but are substantially more pronounced.

### Lyso-PAF alterations in healthy aged controls and AD patients indicate similar mechanisms for all lyso-choline phospholipids

We chose to analyze lyso-PAF (1-alkyl-*sn*-glycero-3-phosphocholine; Fig. 3A and Supplementary Figure 3), another ether-linked phospholipid and a potent inflammatory mediator, as an additional marker for choline phospholipid changes in the plasma of aging control subjects and AD patients (comparison of baseline levels: *p* = 0.003; Fig. 3B). Like most other lipids in our data set, compared with baseline lyso-PAF levels increased moderately (by 20%), but statistically highly significantly, in the control group over the 7.5-year period (ANCOVA: F = 32.95, *p* < 0.001; Fig. 3C). In the probable AD group, the increase was much more prominent with lyso-PAF levels of 151% in females and 203% in males relative to baseline (mean increase across genders: aging group: 20%, AD group: 66%; group comparison using ANCOVA: *p* < 0.001; Fig. 3B,C). Again, the different lyso-PAF subspecies behaved similarly although the rise in the C16:0 and C18:1 subspecies was stronger than for C18:0 (Fig. 3D), which differed only in male AD patients.

To examine, if the observed alterations in the different phospholipid species were correlated, we performed an analysis across all individuals of the study (Supplementary Figure 4). Indeed, we found a moderate correlation (*r* = 0.65, *p* < 0.001) between lysoPC and lyso-PAF levels and a weak correlation (*r* = 0.40, *p* < 0.001) between PlsCho and lyso-PAF levels, indicating that the levels of the different choline-containing lipids appear to be associated.

### Changes in choline-containing phospholipid levels in AD patients are more pronounced than in the normally aging population

**Fig. 4:**
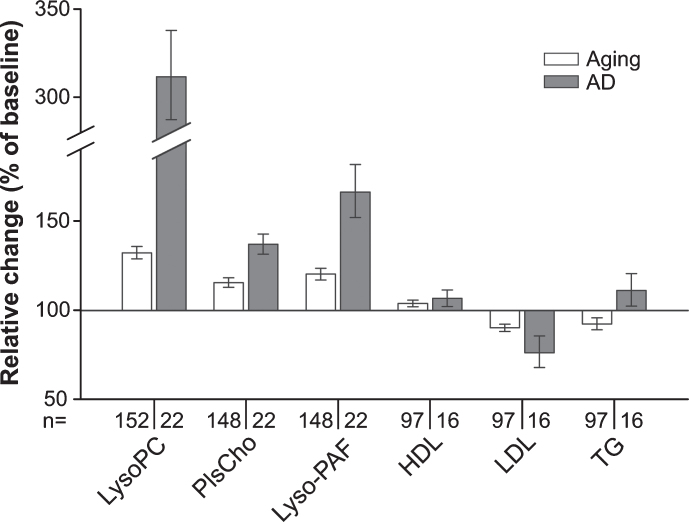
Summarized changes in the plasma levels of choline phospholipids and standard lipid parameters upon aging and conversion to AD. Relative (intra-individual) plasma levels of total lysoPC, PlsCho and lyso-PAF, as well as of HDL, LDL, and triglycerides (TG) at 90-months follow-up (aged controls) or at diagnosis of probable AD after 30, 60, or 90 months (AD patients) are depicted in comparison with the values at baseline (76 years, 100%). Note that in this graph, data for males and females are combined in the control and the AD patient groups. Subjects using lipid-lowering medication at either one or both time points of sampling were excluded from the analysis of HDL, LDL, and TG levels.

We observed that over time the plasma levels of all the measured phospholipid species in the group converting to probable AD shifted in the same direction as in the control group of normally aging individuals. However, the alterations were more pronounced in the AD patients (Fig. 4) in spite of the slightly lower average age of patients at the final sampling. This applied also to plasma lipoprotein levels (Fig. 4 and Supplementary Figure 5); whereas HDL levels increased marginally in both controls and patients developing probable AD, LDL decreased slightly in the control group and more strongly in the patient group. Plasma triglyceride levels changed slightly but in opposing directions in the two groups (Fig. 4 and Supplementary Figure 5).

## DISCUSSION

The present report represents the first longitudinal study of the plasma levels of choline-containing phospholipids in a birth cohort of elderly people. Our results in plasma do not reflect a systemic impairment of peroxisomes in AD, which would have been one of the possible explanations for the peroxisomal alterations observed in our previous study of the brain of AD patients [16]. Levels of lysoPCs containing very long-chain fatty acids were not more increased than other lysoPC species and the levels of ether-linked choline phospholipids, whose biosynthesis is dependent on peroxisomes, were increased rather than decreased. However, our results show consistently that over the examined critical period of aging the plasma levels of lysoPC, PlsCho, and lyso-PAF increase upon normal aging and, more pronounced, upon conversion to probable AD. Several previous studies have investigated in detail changes in the metabolism of choline-containing phospholipids in the brain or cerebrospinal fluid during aging and upon development of AD [30, 32–35]. It is, however, not fully established how phospholipid levels of brain and peripheral blood are related [36] and, therefore, uncertain to what extent findings derived from these studies can be compared with our results. To date, there are only few publications on choline phospholipids in plasma or serum in the context of normal aging or AD. Recent publications report significant increases in a variety of lysoPC species in a middle-aged cohort during a 3-year period [37], in good agreement with our data, as well as increased lysoPC levels in individuals carrying mutations in *PSEN1,* associated with familial AD [38]. Also, two lysoPC species were found increased in the plasma of demented subjects in another study published lately [39]. On the other hand, in contrast to the robust increase that we observed, decreased plasma levels of lysoPC have been described in AD patients in several publications [12, 13, 40]. Interestingly, the lysoPC species with an 18:2 fatty acyl chain has been proposed as constituent of a biomarker panel predicting the conversion to AD [7]. In that study, the levels of lysoPC 18:2 were reduced at baseline in future converters, but tended to increase after conversion to AD [7, 41], reminiscent of our observations. However, whereas our results demonstrate elevated levels after diagnosis of probable AD compared with non-demented controls, the values reported by Mapstone and colleagues were below those of age-matched cognitively normal controls even after AD diagnosis [7, 41]. Of note, a recent cross-sectional investigation aiming to replicate the findings by Mapstone and collaborators indicated that the levels of these proposed biomarkers may be heavily dependent on the study population [42]. In that study, increased lysoPC 18:2 was associated with lower MMSE scores, consistent with our findings.

In most of the previous investigations of lysoPC levels in blood, the mean age of participants was lower than in the VITA study [12, 40]. This difference could be an important determinant, since trends in blood lipid levels can reverse with increasing age. For example, LDL levels continuously rise after adolescence, but decline from the age of about 60 and 70 years in men and women, respectively [43–45]. This was also confirmed by our findings in the normal aging group at 75 and 83 years of age (Fig. 4). In some studies, the mean age differs substantially between AD patients and controls. However, our results indicate that stringent age matching is a crucial factor when evaluating plasma phospholipid levels in health and disease conditions, especially in the critical period for onset of AD. Moreover, we analyzed samples of patients, who had recently converted to probable AD, i.e., at the time point of diagnosis, whereas many other reports do not provide any information on the disease history. This should be considered as an additional reason for the observed differences between the studies, given that long-term disease may have a different impact on plasma lipids than earlier stages of AD. However, the long time span of our study required a rather large follow-up interval (30 months). Consequently, conversion to AD might have taken place months before examination, potentially accounting for differences in the results between our study and Mapstone et al. [7]. Such a temporal factor constitutes a viable explanation for the stronger rise in lysoPC levels from baseline to diagnosis in our investigation compared with Mapstone et al. [7]. In addition, the plasma phospholipid profile is considerably influenced by the amount and type of food intake [46, 47]. Thus, for lysoPC—or the other phospholipid classes—we cannot exclude a contribution from dietary habits to the observed discrepancies, since these likely differ between Central Europeans (our study) and the Asian [13, 40], Mediterranean [12], or North American [7] populations of other studies. We assume variations originating from the diet to be relatively low within the VITA study, as all participants lived in a small geographical area within Vienna. In contrast to the VITA study and [7], the other studies of lysoPC levels were cross-sectional. The longitudinal design may have allowed identification of more subtle changes, which might have been overlooked in the cross-sectional setting.

Concerning the levels of choline ether phospholipids in aging or in AD, apart from a few studies describing decreased amounts of PlsCho in the AD brain [48, 49], scant previous information was available. Interestingly, one study reported PlsCho in the serum of elderly people to be less abundant than in a young cohort [50]. However, we found that the plasma levels of PlsCho increase from the age of 75 to 83 years, again stressing the importance of age when discussing phospholipid alterations in the blood. We again observed a markedly stronger increase in PlsCho upon conversion to probable AD. At the first glance, this outcome appears to be in discrepancy with a metabolomics study, in which serum PlsCho levels of AD patients were mostly unchanged or, by trend, decreased [12]. Our clear-cut results, though, are predominantly due to the longitudinal design of the VITA study following intra-individual lipid profiles. By simply comparing the mean values of the non-demented aging group and the AD group (like in cross-sectional studies), our data also do not reveal statistically significant differences for total PlsCho levels. Supporting these considerations, a recent cross-sectional study reported a trend toward increased levels of several PlsCho species in plasma from early and moderate AD subjects but none of the alterations reached statistical significance [51]. Remarkably, the levels of ether-linked choline phospholipids (which may or may not represent plasmalogens) have also been positively correlated with cerebrospinal fluid biomarkers of AD, particularly A*β*_1 - 42_, and high baseline levels of these lipid species are related to poorer longitudinal cognitive performance [52]. Increased serum PlsCho levels have been associated with an anti-inflammatory status and positively correlated with HDL [53], in line with the slight increases in HDL that we detected during aging as well as in the AD cohort (Fig. 4).

In addition to elevated levels of lysoPC and PlsCho, we also found an accumulation of lyso-PAF in the course of aging, which again was more striking upon development of probable AD. Lyso-PAF is a precursor as well as a degradation product of PAF, a versatile molecule serving as mediator of inflammation [54]. PAF concentrations in the plasma have previously been shown to increase with age [55]. Notably, a recent report described strongly elevated levels of PAF as well as lyso-PAF in the cortex of AD patients, implicating increased hydrolysis of PAF as a means to prevent its detrimental accumulation [56]. Similar mechanisms may contribute also to our findings in plasma.

In conclusion, our results indicate that the conversion to AD is accompanied by marked changes in choline-containing plasma phospholipids, in particular the lyso-forms and choline plasmalogens. These lipids could potentially serve as biomarkers for the diagnosis and, as suggested previously for individual subspecies, even for the prediction of AD. However, we also emphasize that the same lipids are substantially affected by the normal aging process, possibly due to a combination of biological factors and changes in lifestyle or general health status often occurring around the age of 80 years. The suggested use of these lipids as biomarkers is further complicated by the fact that altered levels of lysoPC or phospholipids in general have also been described in other neurodegenerative diseases like stroke or multiple sclerosis [57–59]. Based on our findings, blood-based lipid biomarker candidates appear to be valid within a defined age range, but may vary substantially between different age groups and, thus, might be most predictive when applied as a profile over time. Nevertheless, our results should serve as a note of caution for other studies and particularly implicate age as a highly critical component, which needs to be carefully considered in the search and interpretation of biomarkers.

## Supplementary Material

Supplementary Material
