# Supplementary material for: Alterations in the Plasma Levels of Specific Choline Phospholipids in Alzheimer’s Disease Mimic Accelerated Aging
Source: J Alzheimers Dis. 2018 Feb 20;62(2):841–54. doi: 10.3233/JAD-171036 (PMC5837024; doi:10.3233/JAD-171036)
Supplement: Supplementary Material [file jad-62-jad171036-s001.pdf]

Supplementary Information

Alterations in the plasma levels of specific choline phospholipids in Alzheimer's disease mimic accelerated aging

Fabian Dorninger, Ann B. Moser, Jianqiu Kou, Christoph Wiesinger, Sonja Forss-Petter, Andreas Gleiss, Margareta Hinterberger, Susanne Jungwirth, Peter Fischer, Johannes Berger

Supplementary Table 1: Blood biochemical parameters of the study population

		Aging group			AD group		
	Reference range	All (n=152)	Female (n=94)	Male (n=58)	All (n=22)	Female (n=15)	Male (n=7)
**Baseline**							
Age [years]		75.64 (75.32-75.98)	75.59 (75.30-75.88)	75.73 (75.43-76.03)	75.79 (75.44-76.22)	75.81 (75.49-76.22)	75.45 (75.32-76.22)
Aβ42 plasma [pg/ml]	n.a.	21.5 (15.0-31.0)	22.0* (16.0-31.5)	18.0* (14.0-30.0)	19.5 (13.8-31.8)	18.0 (14.0-27.0)	31.0 (12.0-52.0)
Total cholesterol [mg/dl]	30-200	236 (202-258)	243 (218-276)	214 (184 -239)	233 (201-263)	237 (220-270)	198 (155-239)
LDL [mg/dl]	<130	149 (120-175)	154 (127-183)	137 (110-161)	146 (126-176)	150* (137-180)	124 (87-146)
HDL [mg/dl]	M: 35-130<br>F: 45-130	57 (44-67)	63 (53-73)	47 (40-58)	59 (45-71)	60 (48-70)	56 (32-73)
Triglycerides [mg/dl]	50-150	119 (93-167)	118 (95-159)	133 (85-187)	120 (88-175)	135 (105-177)	87 (67-96)
TSH [µlU/ml]	0.25-4.00	1.06 (0.63-1.68)	1.10 (0.62-1.80)	0.97 (0.67-1.60)	1.41 (0.53-2.67)	0.89 (0.25-2.17)	1.77 (1.40-2.86)
ASAT (GOT) [U/l]	M: 1-18<br>F: 1-15	10.0 (9.0-12.0)	10.0 (9.0-12.0)	11.0 (9.0-12.3)	10.0 (9.0-11.3)	10.0 (9.0-11.0)	10.0 (9.0-12.0)
LDH [U/l]	50-240	177 (164-203)	181 (168-205)	173 (158-184)	172 (152-215)	186 (163-229)	151 (140-188)
Gamma-GT [U/l]	M: 6-28<br>F: 4-18	15.5 (11.0-24.8)	13.0 (10.0-21.3)	18.5 (13.8-25.3)	15.5 (10.8-17.0)	16.0 (10.0-17.0)	15.0 (11.0-19.0)
Total protein [g/l]	60-80	74 (71-78)	75* (71-79)	74.0 (71-78)	75 (72-80)	74.0 (70-80)	77 (73-80)
Creatinine [mg/dl]	M: 0.5-1.2<br>F: 0.5-1.0	1.10 (1.00-1.20)	1.00 (0.90-1.10)	1.20 (1.10-1.30)	1.05 (0.98-1.20)	1.00 (0.90-1.10)	1.10 (1.10-1.40)
Hemoglobin [g/dl]	M: 13-17<br>F: 12.5-16	14.3 (13.6-15.5)	14.0* (13.4-14.7)	15.2 (14.2-16.1)	14.8 (13.7-15.1)	14.2 (13.4-15.0)	15.0 (14.9-15.9)
HbA1C [% Hb]	3.0-6.0	5.7 (5.4-6.2)	5.8 (5.4-6.2)	5.7 (5.3-6.2)	5.7 (5.3-6.4)	5.7 (5.4-6.5)	5.7 (5.2-5.8)
Glucose [mg/dl]	50-110	102 (93-121)	99 (92-111)	109 (97-128)	102 (96-131)	104 (97-138)	98 (96-112)
Fibrinogen [mg/l]	180-350	372 (322-429)	385 (330-439)	365 (313-402)	379 (322-437)	375 (331-411)	434 (258-438)
C-reactive protein [mg/l]	<5	2.0 (0.0-5.0)	2.0 (0.0-5.0)	3.0* (0.0-5.0)	1.5 (0.0-8.0)	2.0 (0.0-8.0)	0.0 (0.0-8.0)
Folate [ng/ml]	3-17	8.9 (6.9-11.6)	9.9 (7.8-12.3)	7.6 (6.0-10.7)	7.5 (4.9-9.4)	8.2 (5.2-11.2)	5.4 (3.8-7.5)
Vitamin B12 [pg/ml]	200-950	429 (333-603)	446 (347-668)	400 (309-521)	442 (359-678)	552 (358-700)	431 (371-523)

Supplementary Table 1 continued

		Aging group			AD group		
	Reference range	All (n=152)	Female (n=94)	Male (n=58)	All (n=22)	Female (n=15)	Male (n=7)
**Follow-up**							
Age [years]		83.22 (82.95-83.52)	83.19 (82.95-83.49)	83.26 (82.96-83.57)	78.85 (78.08-80.92)	79.23 (78.68-81.35)	78.03 (77.91-79.07)
Aβ42 plasma [pg/ml]	n.a.	n.d.	n.d.	n.d.	n.d.	n.d.	n.d.
Total cholesterol [mg/dl]	30-200	205 (176-239)	216 (194-250)	187 (159-213)	215 (183-249)	217 (190-247)	213 (162-253)
LDL [mg/dl]	<130	121 (97-149)	127 (102-156)	109 (88-131)	118 (93-146)	117 (100-142)	134 (77-159)
HDL [mg/dl]	M: 35-130 F: 45-130	59 (49-69)	65 (55-75)	53 (42-60)	61 (51-72)	61 (55-74)	61 (39-66)
Triglycerides [mg/dl]	50-150	108 (86-144)	112 (91-140)	105 (79-161)	126 (97-164)	124 (98-161)	148 (65-172)
TSH [µIU/ml]	0.25-4.00	1.45 (0.99-2.20)	1.50 (1.00-2.23)	1.35 (0.93-2.13)	1.20 (0.49-2.20)	1.10 (0.23-2.00)	1.80 (0.84-2.20)
ASAT (GOT) [U/l]	M: 1-18 F: 1-15	10.0 (9.0-12.0)	10.0[‡] (9.0-12.0)	11.0 (9.0-13.0)	10.0 (8.8-12.0)	9.0[§] (8.0-10.0)	11.0 (9.0-15.0)
LDH [U/l]	50-240	195 (176-223)	195[‡] (179-224)	194* (169-221)	177 (165-211)	203[§] (173-217)	169 (149-175)
Gamma-GT [U/l]	M: 6-28 F: 4-18	n.d.	n.d.	n.d.	n.d.	n.d.	n.d.
Total protein [g/l]	60-80	72 (69-76)	72 (70-76)	72 (69-77)	73 (71-75)	73 (69-75)	73 (72-75)
Creatinine [mg/dl]	M: 0.5-1.2 F: 0.5-1.0	1.10 (0.90-1.20)	1.00 (0.80-1.13)	1.20 (1.00-1.40)	0.90 (0.90-1.10)	0.90 (0.80-0.90)	1.10 (1.00-1.10)
Hemoglobin [g/dl]	M: 13-17 F: 12.5-16	13.8 (12.9-15.0)	13.7 (12.8-14.4)	14.7 (13.1-15.9)	14.3 (13.6-15.6)	13.8 (12.7-14.4)	15.7 (15.0-15.8)
HbA1C [% Hb]	3.0-6.0	6.0 (5.7-6.3)	6.0* (5.7-6.3)	6.0* (5.7-6.5)	5.8 (5.4-6.6)	6.0 (5.4-6.9)	5.6 (5.3-6.6)
Glucose [mg/dl]	50-110	n.d.	n.d.	n.d.	n.d.	n.d.	n.d.
Fibrinogen [mg/l]	180-350	420 (309-506)	440[§] (326-526)	376[†] (287-499)	371 (325-426)	371* (320-419)	364* (308-553)
C-reactive protein [mg/l]	<5	2.0 (1.0-4.0)	2.0 (1.0-4.0)	2.0 (1.0-3.0)	1.0 (0.0-6.0)	2.0 (1.0-6.0)	0.0 (0.0-1.0)
Folate [ng/ml]	3-17	7.6 (5.2-16.3)	8.6 (5.8-18.0)	6.7 (4.8-11.9)	8.4 (5.0-13.0)	9.7 (6.5-14.0)	4.4 (3.2-8.1)
Vitamin B12 [pg/ml]	200-950	358 (256-527)	383 (263-637)	312 (228-442)	478 (321-767)	596 (359-838)	335 (299-547)

Abbreviations: F, female; M, male; LDL, low density lipoprotein cholesterol; HDL, high density lipoprotein cholesterol; TSH, thyroid stimulating hormone; ASAT (GOT), aspartate aminotransferase (glutamyl oxaloacetic transaminase); LDH, lactate dehydrogenase; Gamma GT, gamma glutamyl transferase; HbA1c, glycated hemoglobin

All values are presented as medians with quartiles in parentheses. Follow-up refers to 90 months in the aging group and the time point of diagnosis in the AD group.

* 1 missing value

[†] 2 missing values

[‡] 3 missing values

[§] 4 missing values

Supplementary Figure 1

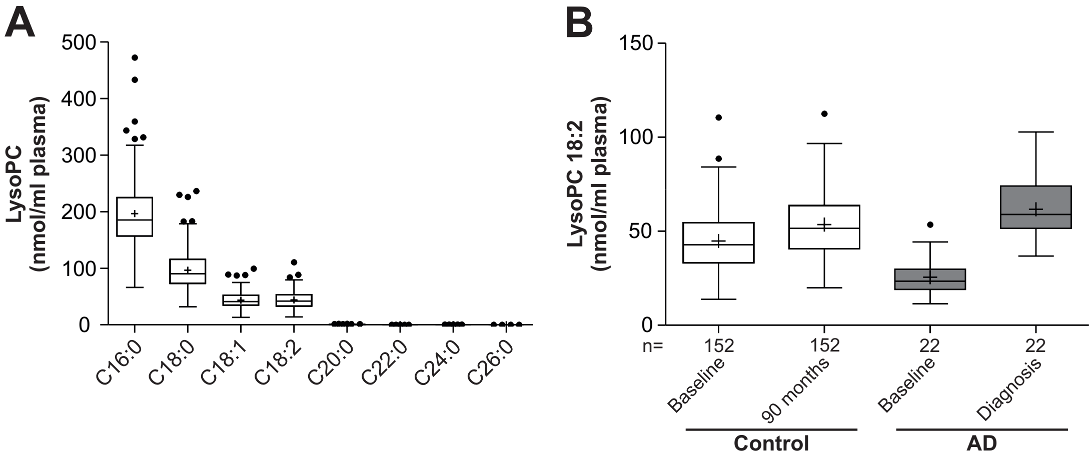

Supplementary Figure 1. ***Supporting information on plasma lysoPC levels.*** (A) Box-and-whisker plots indicate the absolute levels of lysoPC subspecies (identified by the *sn*-1 chain) in control subjects at baseline (76 years, n=152). (B) The distribution of the plasma values of lysoPC with C18:2 as *sn*-1 chain at baseline (76 years) and after 90 months (aged controls) or at diagnosis of probable AD after 30, 60 or 90 months (AD patients) are depicted as box-and-whisker plots according to Tukey's method. The horizontal line inside the box indicates the median value, while + indicates the mean value.

Supplementary Figure 2

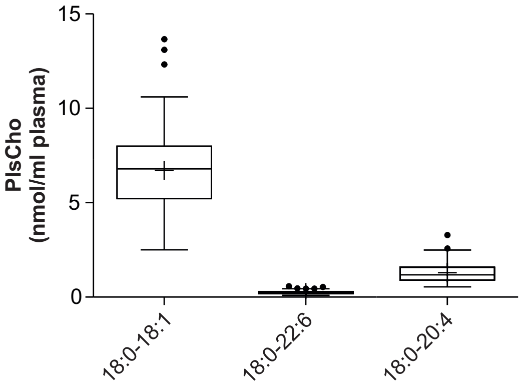

Supplementary Figure 2. ***Supporting information on plasma PlsCho levels.*** Box-and-whisker plots indicate the absolute levels of PlsCho subspecies (identified by the *sn*-1 and *sn*-2 chains) in control subjects at baseline (76 years, n=148). The horizontal line inside the box indicates the median value, while + indicates the mean value.

Supplementary Figure 3

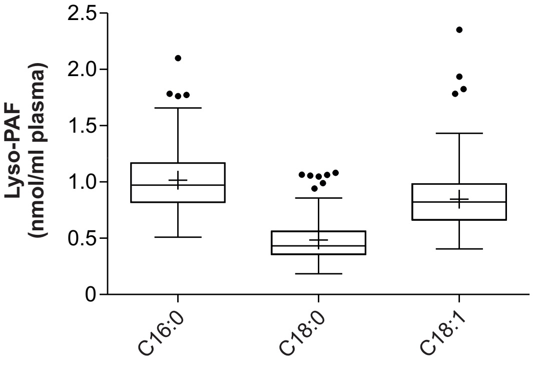

Supplementary Figure 3. ***Supporting information on plasma lyso-PAF levels.*** Box-and-whisker plots indicate the absolute levels of lyso-PAF subspecies (identified by the *sn*-1 chain) in control subjects at baseline (76 years, n=148). The horizontal line inside the box indicates the median value, while + indicates the mean value.

Supplementary Figure 4

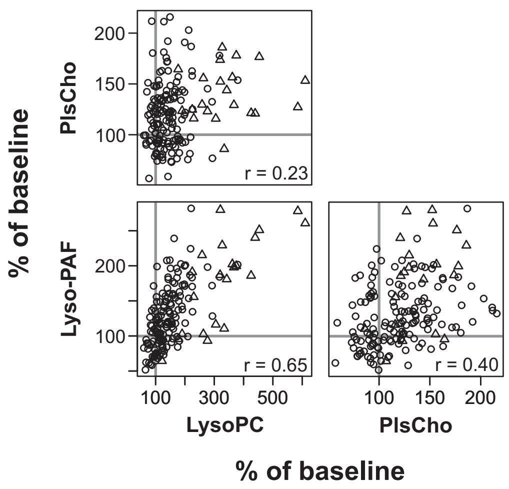

Supplementary Figure 4. ***Correlation analysis of different choline-derived phospholipid species.*** The correlation matrices show the plasma levels of choline-derived phospholipid species in all samples analyzed (baseline and follow-up samples). Circles indicate samples from controls, triangles those from AD patients. Associations were calculated using Spearman's correlation coefficient.

Supplementary Figure 5

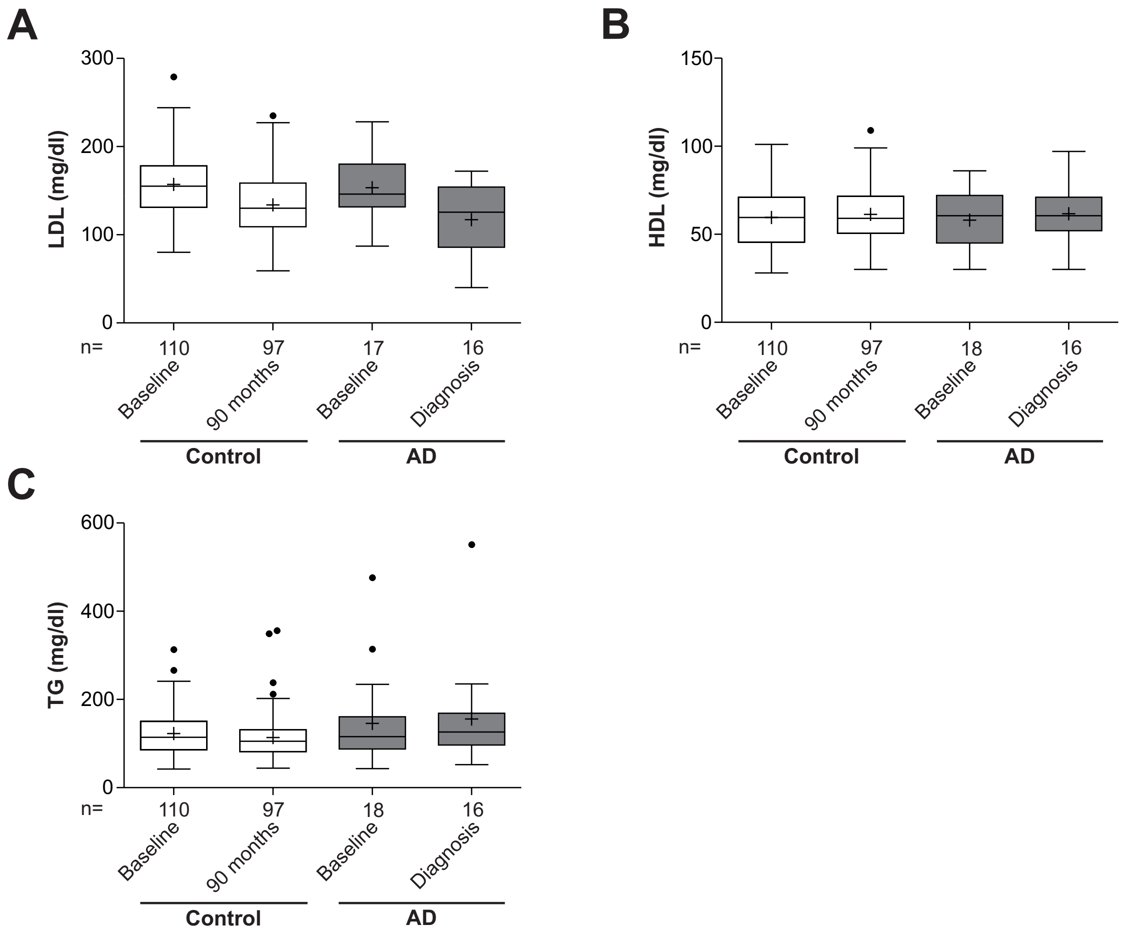

Supplementary Figure 5. ***Supporting information on standard lipid parameters.*** The distribution of the absolute plasma levels of LDL (A), HDL (B) and triglycerides (TG) (C) at baseline (76 years) and after 90 months (aged controls) or at diagnosis of probable AD after 30, 60 or 90 months (AD patients) are depicted as box-and-whisker plots according to Tukey's method. The horizontal line inside the box indicates the median value, while + indicates the mean value. Subjects taking lipid-lowering medication at either one or both time points were excluded from the analysis.
